# A new protocol for investigating visual two-choice discrimination learning in lizards

**DOI:** 10.1007/s10071-022-01603-x

**Published:** 2022-02-06

**Authors:** Birgit Szabo, Martin J. Whiting

**Affiliations:** 1 School of Natural Sciences, Macquarie University, Sydney, Australia; 2 Division of Behavioural Ecology, Institute of Ecology and Evolution, University of Bern, Wohlenstrasse 50a, 3032 Bern, Switzerland

**Keywords:** Associative learning, Cognition, Reptile, Response conditioning, Squamate, Target training

## Abstract

One of the most widely studied abilities in lizards is discrimination learning. The protocols used to test lizards are often novel or adapted from other taxa without proper validation. We need to ensure that tests of discrimination learning are appropriate and properly applied in lizards so that robust inferences can be made about cognitive ability. Here, we present a new protocol for testing lizard discrimination learning that incorporates a target training procedure, uses many daily trials for efficiency and reinforcement, and has a robust, validated, learning criterion. We trained lizards to touch a cue card using operant conditioning and tested lizards separately on a colour, and pattern discrimination test. Lizards successfully learnt to touch a cue card and to discriminate between light and dark blue but had issues discriminating the patterns. After modifying the test procedure, some lizards reached criterion, revealing possible issues with stimulus processing and interference of generalisation. Here, we describe a protocol for operant conditioning and two-choice discrimination learning in lizards with a robust learning criterion that can help researcher better design future studies on discrimination learning in lizards.

**Supplementary Information:**

The online version contains supplementary material available at 10.1007/s10071-022-01603-x.

## Introduction

Animals routinely need to discriminate between stimuli they encounter in the environment (Shettleworth 2010). The ability of animals to learn to discriminate between different stimuli is an intensively studied area of research (Shettleworth 2010). Early research aimed to understand how animals learn to discriminate between stimuli and what information they focus on in the process (Krechevsky 1932; Mackintosh 1965; Spence 1940). In its' most simple version, discrimination learning is tested by presenting animals with two stimuli, of which choosing one results in a reward while choosing the other is not rewarded and, sometimes, incorrect choices are even punished (Shettleworth 2010). Extensions from this basic method include the use of stimuli with multiple features to test executive functions (e.g. Bissonette and Powell 2012; Graf and Tighe 1971; Roberts et al. 1988), stimuli which show similar features to test generalisation (e.g. Avarguès-Weber et al. 2010; Astley and Wasserman 1992; Herrnstein 1979) and multi-stage scenarios aiming to test, for example, behavioural flexibility (e.g. Bissonette and Powell 2012; Clark et al. 2014; Graf and Tighe 1971; Lucon-Xiccato and Bisazza 2014; Szabo et al. 2018; Tebbich and Teschke 2014) or timing/learning strategy (e.g. McMillan et al. 2015; Zentall 2020; Zentall et al. 2020).

An increasing amount of research in comparative cognition is conducted on non-conventional model species such as lizards (Szabo et al. 2021a). Lizards show significant diversity in habitat use, mating system (from monogamy to polygynandry), feeding ecology (insectivorous, omnivorous, herbivorous; sit-and-wait vs active foraging), sociality (solitary to large groups, including family groups), reproductive mode (oviparous, viviparous) and parental care (Halliwell et al. 2017; Pianka and Vitt 2003; Reilly et al. 2009; Shine 1985, 1987; Somma 2003; Waters et al. 2017; While et al. 2014; Whiting and While 2017). Consequently, lizards are a powerful system for studying comparative cognition and behaviour. Closely related species can differ substantially in common traits, while conversely, distantly related species can show high similarity (i.e. convergence, e.g., Kolbe et al. 2011; Ord et al. 2013; Pianka and Vitt 2003). Additionally, they possess a very different brain structure compared to mammals and birds (Nomura et al. 2013). As such, lizards make good models to test a range of topics including learning. Lizards are able to learn to discriminate based on luminance (e.g. Gaalema 2011), chromatic contrast, pattern, or shapes (e.g. Day et al. 1999; Leal and Powell 2012; Qi et al. 2018; Szabo et al. 2018, 2019a, b, 2021b; Szabo and Whiting 2020), location (e.g. Batabyal and Thaker 2019; Noble et al. 2012) and also show proficiency in reversal learning (e.g. Batabyal and Thaker 2019; Clark et al. 2014; Day et al. 1999; Gaalema 2011; Leal and Powell 2012; Noble et al. 2012; Szabo et al. 2018, 2019a, 2021b; Szabo and Whiting 2020).

The earliest research into lizard discrimination learning aimed to investigate visual perceptual ability (e.g. Benes 1969; Ehrenhardt 1937; Vance et al. 1965; Wagner 1933). This focus eventually shifted (e.g. Day et al. 1999; Loop 1976; Shafir and Roughgarden 1994) and now researchers mainly use discrimination learning tests to study lizard learning exclusively in the visual domain (e.g. Bezzina et al. 2014; Damas-Moreira et al. 2018; Gaalema 2011; Leal and Powell 2012; Munch et al. 2018; Noble et al. 2014; Qi et al. 2018; Riley et al. 2018; Szabo et al. 2018; 2019a; b; 2021b; Szabo and Whiting 2020; Whiting et al. 2018). While testing lizards for many hundreds of trials presenting stimuli around 5–6 trials per day was the norm in early studies (Benes 1969; Vance et al. 1965; Loop 1976; Shafir and Roughgarden 1994), we found a trend towards presenting stimuli only a few times per day in most recent studies (1–3 trials per day; Bezzina et al. 2014; Clark et al. 2014; Damas-Moreira et al. 2018; Leal and Powell 2012; Munch et al. 2018; Noble et al. 2014; Qi et al. 2018; Riley et al. 2018; Szabo et al. 2018; 2019a; b; 2021b; Szabo and Whiting 2020; Whiting et al. 2018); with rare exceptions (Gaalema 2011). The reason is often lower food motivation due to the ectothermic nature of lizards, their low metabolism rate, and associated low food intake, which limits the number of trials that can be run per day (Whiting and Noble 2018). Consequently, preferred rewards are used to keep motivation high but with the downside that animals gain weight which can negatively affect their welfare (Benn et al. 2019). In larger lizard species (> 10 cm snout-vent length) smaller rewards can be given frequently while avoiding over-feeding (e.g. Gaalema 2011). When working with small lizard species, however, reward size can quickly reach a lower limit at which point environmental and experimental factors (faster rate of drying, limited small size of insect prey or inability by the researcher to provide a very small reward effectively) make the use of small rewards unfeasible.

Stringent learning criteria in which lizards had to either perform as many as 20 errorless trials before moving on to a new test (Ehrenhardt 1937; Wagner 1933) or had to perform consistently well (e.g. above 80% correct a day) across multiple test days (Benes 1969; Ehrenhardt 1937; Loop 1976; Vance et al. 1965) were common in early studies. Such stringent criteria were necessary because perceptual thresholds were investigated. Unfortunately, these stringent criteria were not consistently adopted when the focus shifted towards studying learning. Contemporary studies of lizard discrimination learning often apply a set learning criterion that can be achieved over multiple days such as 5 out of the last 6 trials correct (Noble et al. 2014; Qi et al. 2018), 6/6 or 7/8 consecutive trials correct (Leal and Powell 2012; Munch et al. 2018; Riley et al. 2018; Szabo et al. 2018; 2019a, b, 2021b; Whiting et al. 2018) or more, following a binomial distribution (Damas-Moreira et al. 2018; Day et al. 1999; Szabo and Whiting 2020). Some rare exceptions use a very stringent criterion (Gaalema 2011) but others did not apply a criterion at all (Bezzina et al. 2014). Any deficiency or inconsistency in testing methodology is concerning because first, without a proper testing procedure we are likely to over- or under-estimate learning and second, a robust procedure will improve data quality and consequently help us better understand the evolution of cognition across taxa through comparative approaches.

Here, we used gidgee skinks (*Egernia stokesii*), a medium sized (15.5–19.0 cm adult snout-vent length; Chapple 2003) Australian lizard species to develop a robust protocol for discrimination learning in lizards in laboratory studies. This species is found in semi-arid areas of western New South Wales to the south-western centre of Western Australia (Cogger 2014). They are active during the day, relatively long-lived (25 years; Chapple 2003) and feed on seeds, fruits and other parts of plants as well as invertebrates depending on season (Duffield and Bull 1998). Gidgee skinks are among the most social lizards. They live in large, stable, and multi-generational family groups comprised of a single monogamous, reproductive pair and their offspring (Duffield and Bull 2002a; Gardner et al. 2001; 2006). Offspring delay reproduction and stay in the family group long after they reach sexual maturity (around 5 years of age; Chapple 2003). Generally, these lizards show high site fidelity and low rates of dispersal (Duffield and Bull 2002b; Gardner et al. 2001). We chose this species because they have previously shown that they are able to learn to discriminate between visual stimuli based on colour and shape in the lab (Szabo et al 2021b). Furthermore, these lizards habituate well to captivity and as an omnivorous, medium-sized species, were repeatedly fed small vegetable pieces in a captive setting while staying motivated for food and without gaining excessive weight in the process. This study had two aims: (1) to develop a protocol that allowed us to test these animals with more than two to three trials per day without them loosing motivation or gaining access weight. (2) Contrary to most contemporary discrimination learning studies in lizards we applied a conservative learning criterion (at least 8/9 correct choices or better in each of two consecutive sessions) which we validated using a reversal session.

First, we developed a target training procedure teaching lizards to approach and touch a target card attached to a wooden apparatus by successive approximation (a step-by-step training procedure to teach complex behavioural sequences). We subsequently used this behaviour in a pilot to test two individuals on a simultaneous two-choice visual discrimination task between a light and dark blue cue and evaluated our learning criterion by giving lizards one reversal trial after they had reached the learning criterion. We then used the developed technique (target training and discrimination procedure) to test six naïve lizards on a simultaneous two-choice visual pattern discrimination. Based on the data from the pilot, we expected our six test lizards to acquire the target-trained behaviour and the pattern discrimination at a similar rate as the two lizards tested in the pilot. However, contrary to our prediction, all six test subjects did not learn the pattern discrimination although all acquired the target behaviour at a similar rate as the lizards tested in the pilot. To find out what was causing the lizards to fail the pattern discrimination we implemented small changes to the testing procedure. These changes resulted in novel insights that have the potential to substantially improve future studies of a similar kind.

## Material and methods

### Animals and husbandry

We used eight adult gidgee skinks (*Egernia stokesii*) of undetermined sex in this study. Lizards were collected from the wild around Fowlers Gap Arid Zone Research Station (− 31.086972 S, 141.704836 E), New South Wales, Australia during March 2018. Individuals were transported by car in cloth bags within a cooler box to Macquarie University, Sydney within a week of capture and were individually housed in plastic tubs (683 L × 447 W × 385 H mm). Lizards were housed in a temperature-controlled environment (24 °C ± 2 SD), with relative humidity between 30 and 60% and a light cycle of 12 h (06:00–18:00 h). In addition to the room lighting, UVB light (URS^®^ Outback Max 10.0 UVA & UVB tube) was provided approximately 800 mm above the enclosure floor. A heat cord underneath one side of the enclosures ensured that animals were able to thermoregulate by increasing the temperature to up to 33 °C (± 2 °C SD), thereby creating a thermal gradient which lizard readily used. iButtons (Thermochron iButton model DS1921) recorded temperature hourly within enclosures. Each enclosure was lined with paper and included a refuge for shelter (upside down, brown plant saucer 200 mm in diameter; 40 mm high), a water bowl (heavy, poly resin reptile water bowl, 130 L × 110 W × 40 H mm) and a wooden ramp, a stone, some bark, leaves, and a 150 mm long PVC tube as enrichment.

Lizards were fed on Monday, Wednesday and Friday with an assortment of small cut fruits (e.g. apple, banana, pear, tomato, strawberry) and vegetables (e.g. carrots, zucchini, capsicum, celery, broccoli, different leafy greens such as lettuce, kale, pak choi, choisum, beet root greens). On Fridays, they received 2–3 crickets powdered with aristopet Repti-vite and URS Ultimate Calcium in addition to the fruits and vegetables. On days on which test sessions were conducted, lizards did not receive their regular diet but were only given food as reward when making a correct choice (1–10 times 0.065 g ± 0.021 SD of carrot) except for Fridays, when they were fed their regular diet (to provide optimal nutrition) as well as any reward obtained during test sessions. On Fridays, animals were fed only after all test sessions were completed. Lizards had ad libitum access to water.

### Setup

All lizards were tested within their home enclosure to avoid stress caused by handling (Langkilde and Shine 2006). Before the start of a test session, a lizard was gently carried (within its enclosure) to a test area approximately 3 m away from the housing area within the same room. Lizards were given 5 min to acclimate before the first test trial started. A grey curtain surrounded the test area and obscured the researcher during trials. Similar to the housing set-up, a heat cord installed under part of the enclosure ensured that lizards were able to thermoregulate during test sessions.

### Apparatus and stimulus cards

**Fig. 1:**
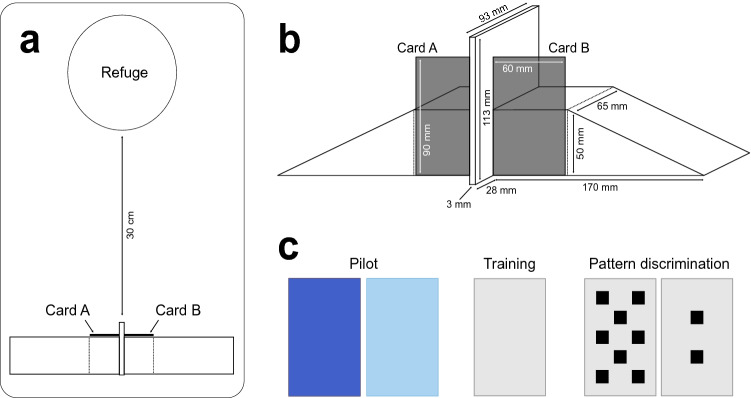
**a** Schematic, top-down view of the set-up used during test trials. The wooden apparatus was placed at one end of the enclosure and the lizard moved back 30 cm under the refuge at the opposite end of the enclosure. Stimulus cards were fixed on each side of the apparatus during discrimination trials. **b** Side view of the wooden apparatus used during test trials. The stimulus cards were attached at the front, to the left and right of a wooden divider using Bostik Blu Tack^®^ adhesive putty preventing them from falling off after being touched by a lizard. **c** Stimuli used during the pilot colour discrimination test (dark and light blue card), target training (light grey card), and the pattern discrimination test (light grey card with either eight or two black squares). Created using Adobe Illustrator 2021 (color figure online)

The wooden apparatus consisted of two wooden ramps (170 L × 65 W × 50 H mm) glued together back-to-back with a wooden coaster (3 mm L × 93  W × 113 H mm) in-between, using non-toxic silicon (Fig. 1b). Each lizard was tested with only its own apparatus to prevent any effect of scent on behaviour. Stimulus cards (60 L × 90 H mm) were created in Microsoft PowerPoint and then printed, laminated, and cut out. The squares depicted on the cards used for the pattern discrimination test were 1 cm^2^ (10 mm × 10 mm) in area. Each lizard received their own sets of cards (i.e. not interchangeable), which were cleaned with 70% ethanol after each session.

### General procedure

Each lizard participated in one session of 10 trials (in the target training: 10 training trials and in the discrimination test: 1 training trial and 9 test trials) per day between 7:30 and 10:30 h, every day for 5 days a week, Monday to Friday. The order in which the test subjects were tested each day was randomised to avoid order effects. First, all enrichment items and the water bowl were removed from the enclosure and the lizard gently covered with the refuge to prevent it from watching the set-up. Next, the lizard was slowly moved as far back as possible while under the refuge (Fig. 1a) and a wooden apparatus (Fig. 1b) was placed at the opposite end of the enclosure nearest to the experimenter. The lizard was left undisturbed under its’ refuge for 30 s before the first stimulus presentation. A trial started by removing the refuge and presenting the lizard with a single stimulus card (target training, Fig. 1c) or two cards attached to the wooden apparatus (discrimination tests, Fig. 1b, c). A trial lasted until the lizard had either touched a card or a maximum of 5 min had elapsed after which the trial was terminated. If a lizard did not touch a card (i.e. make a choice) in two consecutive trials the whole session was terminated. At the end of a trial the lizard was again gently covered by the refuge and moved backwards within the enclosure for an inter-trial interval (ITI) of 30 s.

Carrot strips (created using a grater and then cut into equally sized pieces, 0.065 g ± 0.021 SD each) were used as a reward for a correct response both during target training and discrimination learning. Carrot is a favoured food item for these lizards in captivity (personal observation made during regular husbandry; also see Szabo et al. 2021c) and were prepared fresh each day.

### Target training

Target training was used to teach lizards to touch a target card attached to the wooden apparatus with the goal to use this behaviour in future simultaneous two-choice discrimination tasks (similar target training procedures were used in Hellmuth et al 2012).

**Fig. 2:**
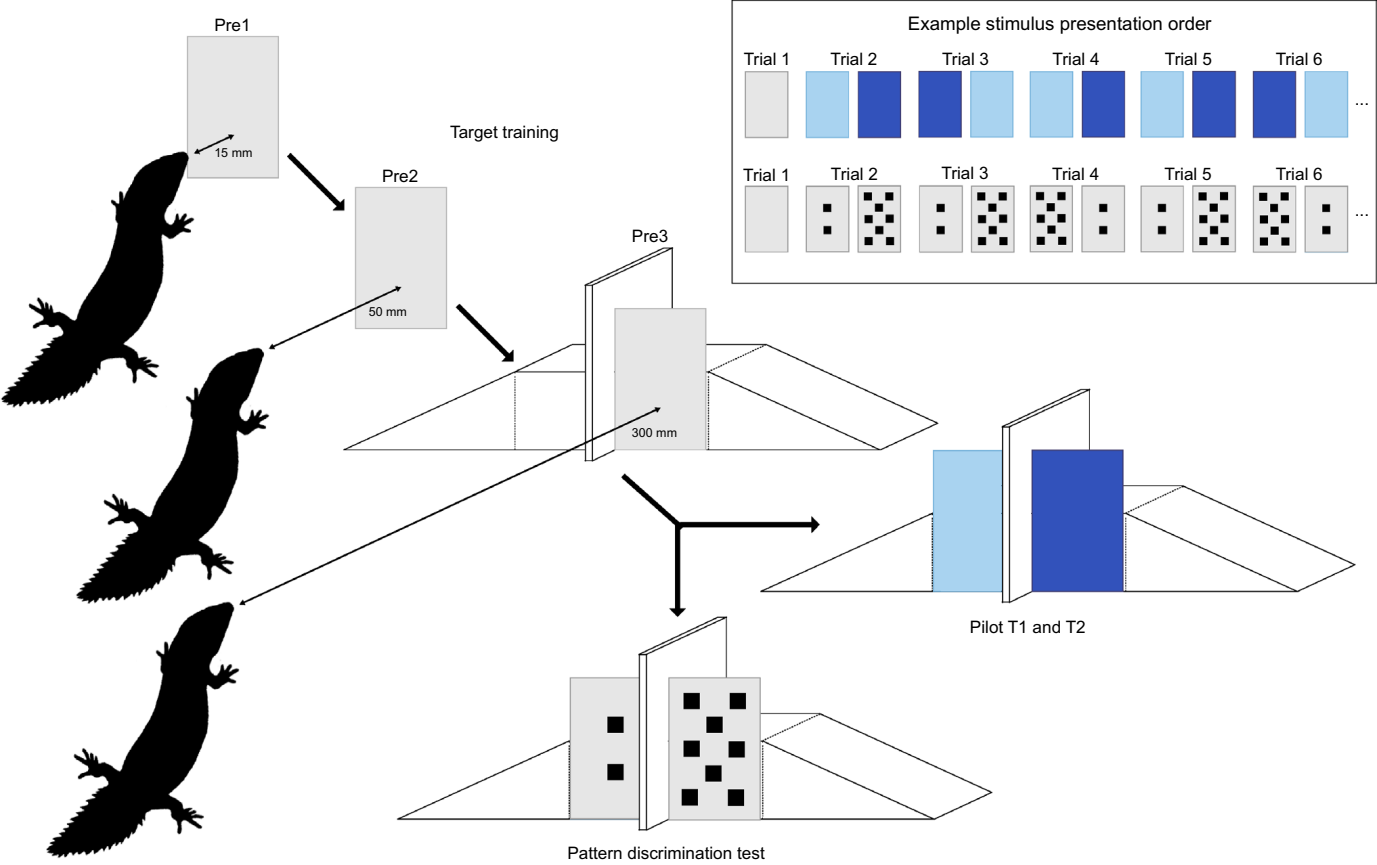
Graphical representation of the training steps to teach the lizards to approach a stimulus card attached to a wooden apparatus (target training) followed by the colour discrimination test (pilot) used for two lizards and the pattern discrimination test (test) used in six lizards. Also included are two examples of possible stimulus presentation order for the first five trials (out of 9) of a session used in the colour as well as the pattern discrimination tests. Trial 1 was always a Pre2 training trial. Pre1—first step of target training in which the cues card was held 15 mm away from the lizard to teach it to touch the card; Pre2—second step of target training in which the cue card was held 50 mm away to teach the lizard to approach and touch the card; Pre3—third step of target training in which the card is fixed to the wooden apparatus similar to the discrimination test; *T1* discrimination test; *T2* reversal test. Created using Adobe Illustrator 2021 (color figure online)

In the first step (Pre1) we taught the lizard to associate touching a card with receiving food (Supplementary Video M1). To this end, we presented the lizard with the single grey stimulus card in front of its head, 15 mm from its snout (Fig. 2) after the refuge was removed at the beginning of a trial. The stimulus card was attached to a pair of forceps using Bostik Blu-Tack^®^ adhesive putty for easy presentation. To initiate approach of the card, the experimenter presented a strip of carrot held in a second pair of forceps directly in front of the stimulus card for 1 s, after which the carrot was hidden behind the stimulus card. This was repeated every 5 s until the lizard touched the card with any body part, which resulted in the lizard receiving the reward. This step was repeated for as many trials as it took until the lizard touched the card without the presentation of the carrot. A lizard moved on to the next step after touching the card without reward presentation in every trial for at least three consecutive sessions (i.e. 30 trials).

The next two steps were designed to teach the lizard to approach the card from a distance. To this end, we presented the stimulus card 50 mm away from its snout (Pre2; Fig. 2; Supplementary Video M1). If the lizard did not approach the card it was shown the carrot, as in the previous step. The criterion to move on was, again, to touch the card without reward presentation in every trial for at least three consecutive sessions (i.e. 30 trials). From this point on, we presented the card 50 mm away from the lizard (Pre2 procedure) in every first trial of a session (in Pre3, colour and pattern discrimination) to keep reinforcing the touching of the stimulus card throughout the whole experiment.

In the third and final step (Pre3), the cue card was held in front of the wooden apparatus (Fig. 2; Supplementary Video M1) from the start of a trial (except for trial 1 in each session) on the left or right side in a predetermined pseudorandom fashion no more than twice consecutively on the same side. Again, if the lizard did not approach the card immediately, the carrot strip was shown. For a lizard to move on to the visual discrimination test, they had to approach and touch the card without reward presentation in every trial for at least three consecutive sessions (i.e. 30 trials).

### Pilot test

We used two lizards in the pilot. In the visual discrimination test (T1), two cards, one light and one dark blue were attached to either side of the apparatus (Figs. 1b, and  2; Supplementary Video M1). Light and dark blue were chosen as stimuli for the pilot because gidgee skinks had shown an ability to discriminate between these two colours in a previous study (Szabo et al 2021b). One of the two lizards used in the pilot was assigned light blue as the correct stimulus, while the other was assigned dark blue as the correct stimulus. Trials were run as follows (except for trial 1 in each session; see above): first, the cards were simultaneously attached to the apparatus after the lizard was already under the refuge. Second, the refuge was removed and the experimenter moved behind the curtain. Third, the experimenter observed the lizards behaviour live on a video screen. Lizards were filmed from above using a CCTV system (3-Axis Day & Night Dome Camera recorded with a H.264 Digital Video Recorder). If the lizard touched the correct card the experimenter emerged from behind the curtain and rewarded the individual with a carrot strip presented in forceps. If, however, the lizard touched the incorrect card, the lizard was covered with the refuge and moved gently to the back of the enclosure in preparation for the next trial (for an ITI of 30 s). If the last choice within a session was incorrect, we conducted another Pre2 trial in which the grey target card was presented 50 mm in front of the lizard to ensure a session ended on a positive note.

Each lizard received one target training (Pre2 procedure) plus nine discrimination trials within one session per day, and had to complete at least three sessions before the learning criterion of 8/9 correct choices or better in each of two consecutive sessions was applied. The side (left/right) that the correct card was presented was predetermined and pseudorandomized to never appear on the same side more than twice in a row. To confirm that a lizard had learned the discrimination they were tested on a reversal session (T2) in which the previously incorrect stimulus became correct and vice versa. The pilot was conducted from the end of April to the beginning of June 2019.

### Pattern discrimination test

We used six naïve lizards to test pattern discrimination using the same training and test procedure developed and verified in the pilot. The whole experiment (including target training and pattern discrimination) was conducted from June to September 2019.

#### Target training (Pre1–Pre3)

We made small changes to the first two target training session to facilitate learning of the target-trained behaviour. In the first five trials of the first training session we presented a carrot strip in forceps to the lizard without the target card then placed the carrot on the enclosure floor (1–2 cm away from the lizard) for the lizard to eat (pre-pre). In the following five trials of the same session, the carrot was presented again in forceps but the lizard had to eat the carrot from the forceps held out by the experimenter (preT). All lizards ate all carrots in the first training session.

In the first five trials of the second training session the target card was presented 15 mm away from the lizard’s snout and the carrot strip was presented in front of the card and not hidden behind the card (Supplementary Video M1). This resulted in the lizard touching the card while eating the carrot. The following 5 trials of the same session were conducted as described above (Pre1).

Finally, instead of holding the target card in front to the apparatus in Pre3 of the training, it was attached to the apparatus from the start of a trial. The rest of the target training was performed exactly as described above (Pre1–Pre3).

#### Pattern discrimination

In the pattern discrimination test, lizards had to learn to discriminate between a grey card depicting two squares and a grey card depicting eight squares. We followed the procedure described above for the visual discrimination test (T1): lizards were each tested in one session of 10 trials (1 training and 9 test trials) per day for 5 days a week until they reached a learning criterion of 8/9 correct choices or better in each of two consecutive sessions (after completing at least three sessions). For three of the six test lizards (randomly chosen) the card depicting two squares was assigned as correct (stimulus group 2), while for the other three lizards the card depicting eight squares was assigned as correct (stimulus group 8). The side a stimulus card was presented was predetermined for each session and followed a pseudorandom order in which the same card was never presented more than twice on the same side. As described above, the first trial of each session was conducted as a Pre2 training trial. This ensured that, even when a lizard made many incorrect choices during test trials (not receiving food for touching a card), they would continue to reliably perform this behaviour throughout the whole experiment.

Based on the data collected in the pilot we expected lizards to acquire the pattern discrimination within approximately 10 sessions (90 trials). However, we did not find the expected performance and decided to implement some minor changes in the test procedure to investigate the reason for the lizards’ poor performance:

1. Starting from the 12th test session, we replaced the single grey card presented in the first trial (Pre2) of each session to reinforce the target behaviour (touching the card) with the stimulus card that was assigned as correct for each lizard (similar to a matching-to-sample test) (Supplementary Video M1). We hoped that reinforcing the correct stimulus card in this way would improve performance, but it did not (see "Results").
2. After the 21st session, we conducted a whole Pre2 session (target training) but we used the correct stimulus card (either showing two or eight squares depending on test group) instead of the empty grey card. For a whole session of 10 trials, we presented the correct card 50 mm away in front of the snout of each lizard reinforcing touching of the stimulus cards with a carrot (all lizards reliably approached and touched the card in all trials without the presentation of the carrot). We hoped that this would further reinforce choosing the correct card, but it did not lead to an improvement in performance (see "Results").
3. Starting from the 32nd test session, we moved the apparatus instead of covering the lizard with the refuge and moving it backwards. We hypothesised that stress might negatively affect the lizards’ performance and wanted to reduce physical handling time. After a lizard had made a choice (correct or incorrect), the apparatus with the stimulus cards attached was slowly lifted out of the enclosure. Only thereafter, was the lizard gently covered with the refuge but not moved. We attached the cards in the configuration needed for the next test trial to the apparatus before placing it back inside the enclosure at the opposite end, furthest away from the lizard (Supplementary Video M1). This change had a significant but small effect on trial choice in one group and a strong effect on latency in both groups (see "Results").
4. Starting from session 42, we stopped cleaning the stimulus cards after each session to facilitate odour accumulation on the correct card because it was touched by the lizard more often than the incorrect card (first trial in each session). This change did not improve the lizards’ performance (see "Results").
5. Finally, in sessions 52–54, we replaced the incorrect card with an empty grey card to increase discriminability between the two stimulus cards. This change had an effect on the lizards’ choice performance (see "Results").

#### Data collection

For the target training we recorded if the reward was shown to the lizard, how often it was shown to the lizard, and if a lizard made a correct response (touching the cards) thereby receiving the reward for each trial. For each test trial (colour and pattern discrimination) we recorded if the response was correct or incorrect (1—correct choice, 0—incorrect choice), the latency to choice (from the removal of the refuge up to the point when a lizard touched a card regardless of if the response was correct or incorrect) in addition to the above described measurements. Furthermore, for each trial we recorded the date a session was performed, the start time of each session, and the initials of the researcher conducting the trial (all trial were conducted by the first author). We also recorded which stimuli were used in each trial (e.g. g—empty grey card, l/r—left or right position of the grey card in Pre3, lb/db—light/dark blue card presented on the left from the experimenters perspective in T1/T2, 2/8—card showing two or eight squares presented on the left in T1). Enclosure temperature was recorded with Thermochron iButtons (model DS1921) and added to the raw datafile based on date after data collection had finished.

### Statistical analyses

We were primarily interested in analysing if any of the five changes we made to the procedure had an effect on lizards’ choice performance and latency to choice. To this end, we assigned a unique letter (b–f, a representing the original procedure used in the first 11 sessions) to the sessions representing a change in procedure (= stages of the test). We used Bayesian generalised linear mixed models (GLMM; R package MCMCglmm, Hadfield 2010) to compare the performance following each change with the previous sessions: stage a was compared to b (Pre2 trial 1 with a card showing the correct stimulus), b compared to c (additional target training with a card showing the correct stimulus), c compared to d (reducing physical contact with the lizards), d compared to e (no cleaning of the cue cards with ethanol), and e compared to f (replacing the incorrect card with an empty grey card).

To analyse choice behaviour of the whole group (not considering stimulus group) we used choice made in each trial (1—correct, 0—incorrect, Bernoulli variable) as the response variable and both stimulus group and stage in interaction with session as the fixed effects. To analyse choice behaviour of each stimulus group, we used choice made in each trial (1—correct, 0—incorrect, Bernoulli variable) as the response variable and the interaction between stage (a–f) and session as the only fixed effect. Session (scaled and centred) was included as a fixed effect because we were not just interested in the overall effect but also in the possible effects on the rate of change (e.g. learning). Additionally, we wanted to know if choice performance increased across all sessions (excluding the last three sessions in which the incorrect cards were replaced). To this end we ran a model with choice made in each trial (1—correct, 0—incorrect, Bernoulli variable) as the response variable and session (scaled and centred) as the only fixed effect. In all models we included a random intercept of ID interacting with a random slope of trial nested in session as the random effect (random intercept and slope model). This way, we were able to account for non-independence and autocorrelation across successive choices (repeated measures of trial and session across individuals).

We ran similar models to analyse differences across stages (a–f) in latency to choice, but instead used the log transformed latency in seconds as the response variable. Log transformation was used because latency data generally are log normal distributed and the DIC of the model using log transformed latency was much smaller than that of the model run without transformation (DIC_non-log_ = 16,650.5; DIC_log_ = 3334.9). Using the posterior of the models we calculated mean estimates and Higher Posterior Density intervals (CIs—confidence intervals) for each stage comparison. We assumed statistical significance if the confidence intervals did not cross 0. Finally, we were interested if performance (choice and latency) were associated with lizard body size or room temperature. We added the lizards SVL (snout-vent length in mm) and room temperature and their interaction as additional fixed effects to the models looking at general patterns across sessions. In all cases, trial 1 (Pre2 trial) was removed before analysis.

As a prior we used a common weak prior (for all models) as we had no specific prior knowledge regarding the lizards’ performance using this testing procedure (for details see R code provided on OSF). We used binomial models with a logit link function when choice was used as the response variable and gaussian models with identity link function when latency was used as the response variable. For all models, we confirmed that no autocorrelation (correlation between lags < 0.1; Hadfield 2010) was present, that sufficient mixing (by visually inspecting plots of MCMC chains; Hadfield 2010) was achieved and that the Markov chain was run for long enough (Heidelberg and Welch diagnostic tests; Hadfield 2010). All analyses were conducted in R version 4.0.3 (R Core Team 2020) and all raw data sets generated during this study and code for analysis are available on the Open Science Framework (10.17605/OSF.IO/SDUX7).

## Results

### Pilot

**Fig. 3:**
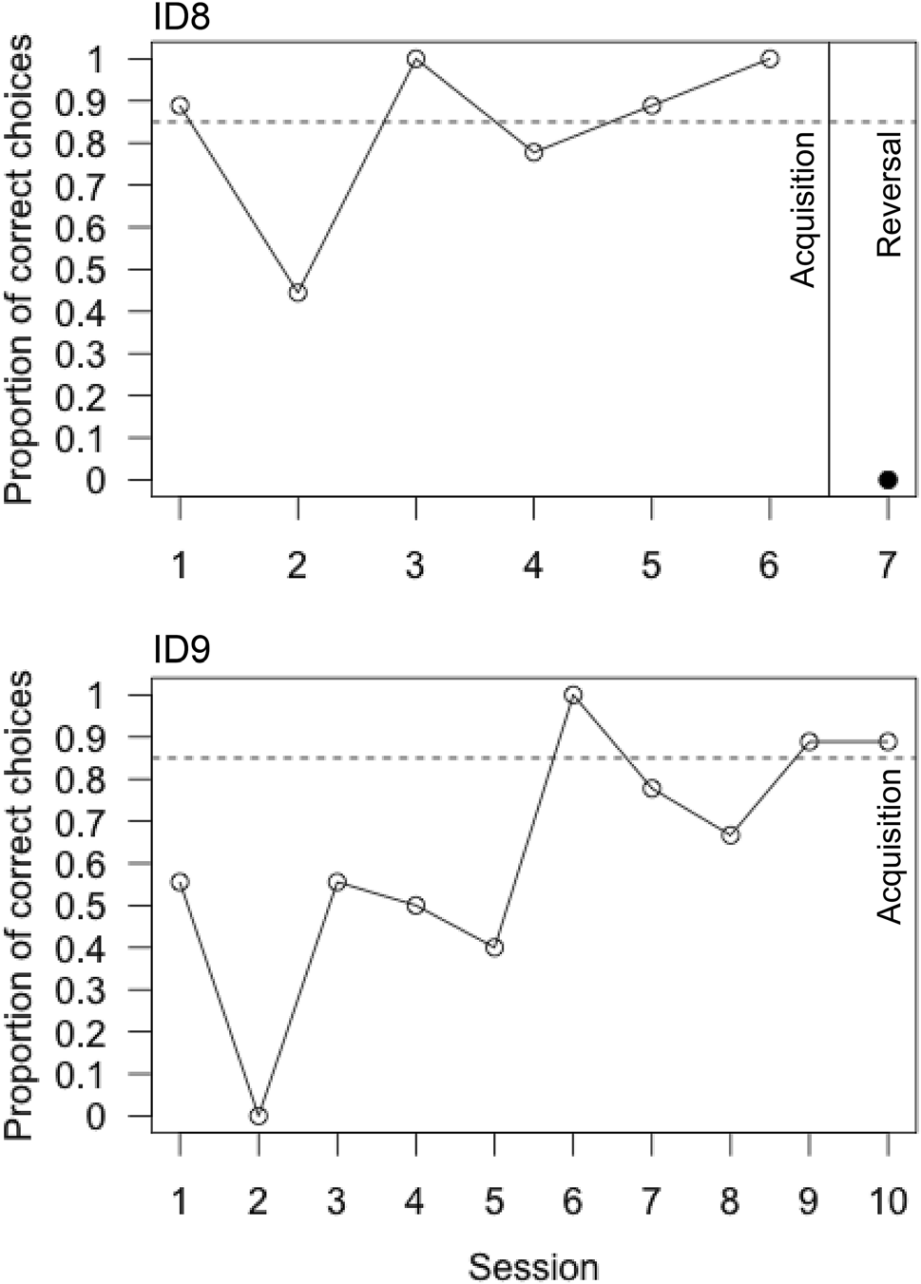
Proportion of correct choices (out of all trials completed) across sessions made during the acquisition (empty circles) of the visual choice discrimination test (Pilot) between a light and dark blue cue card by lizard ID8 (top) and ID9 (bottom) as well as proportion of correct choices made in the reversal session (full circle). Only ID8 completed the entire reversal session, ID9 did not complete a single trial within the reversal session. The horizontal dashed line indicates the learning criterion of 8/9 correct choices or better in each of two consecutive sessions. Created using R base plot and modified using Adobe Illustrator 2021

Results presented for the pilot are only descriptive. No statistical analyses were performed. Lizard ID8 took the minimum of three sessions to reach criterion in Pre1, the minimum of three sessions to reach criterion in Pre2 and the minimum of three sessions to reach criterion in Pre3 (Table 1). Lizard ID9 took five sessions to reach criterion in Pre1, the minimum of three sessions to reach criterion in Pre2 and the minimum of three sessions to reach criterion in Pre3 (Table 1). Lizard ID8 took six sessions to reach the learning criterion in T1 (Table 1, Fig. 3) and made 0 correct choices in the reversal (T2, Fig. 3). Lizard ID9 took 10 sessions to reach the learning criterion in T1 (Table 1, Fig. 3) and did not make a single choice in the reversal trial (T2).

**Table 1:** Average sessions taken by each lizard to reach criterion in the three target training phases (Pre1–Pre3) as well as the pilot and pattern discrimination tests

Test	Pre1	Pre2	Pre3	T1	Animal identity	Snout vent length (mm)
Pilot—LB	3	3	3	**6**	ID8	175
Pilot—DB	5	3	3	**10**	ID9	210
Test—2	4	4	3	53	ID6	186
Test—2	7	3	3	53	ID11	172
Test—2	4	3	3	54	ID16	160
Test—8	5	3	3	**53**	ID4	170
Test—8	4	3	3	**54**	ID7	171.5
Test—8	6	4	3	53	ID10	151

For the pattern discrimination (Test) the number of sessions performed in total is given for each individual. In the test, only lizard ID7 and ID4 reached the learning criterion after 54/53 sessions, respectively. Data for animals that reached the learning criterion are highlighted in bold. LB—light blue as the correct stimulus, DB—dark blue as the correct stimulus, 2—two squares as the correct stimulus, 8—eight squares as the correct stimulus

### Pattern discrimination

All lizards ate all carrot strips presented in the five pre-pre and preT trials. On average lizards took 5 ± 1.27 (mean ± SD) sessions to reach the criterion in Pre1, 3.33 ± 0.52 (mean ± SD) session in Pre2 and 3 ± 0 (mean ± SD) sessions in Pre3 (Table 1).

#### Choice performance

**Fig. 4:**
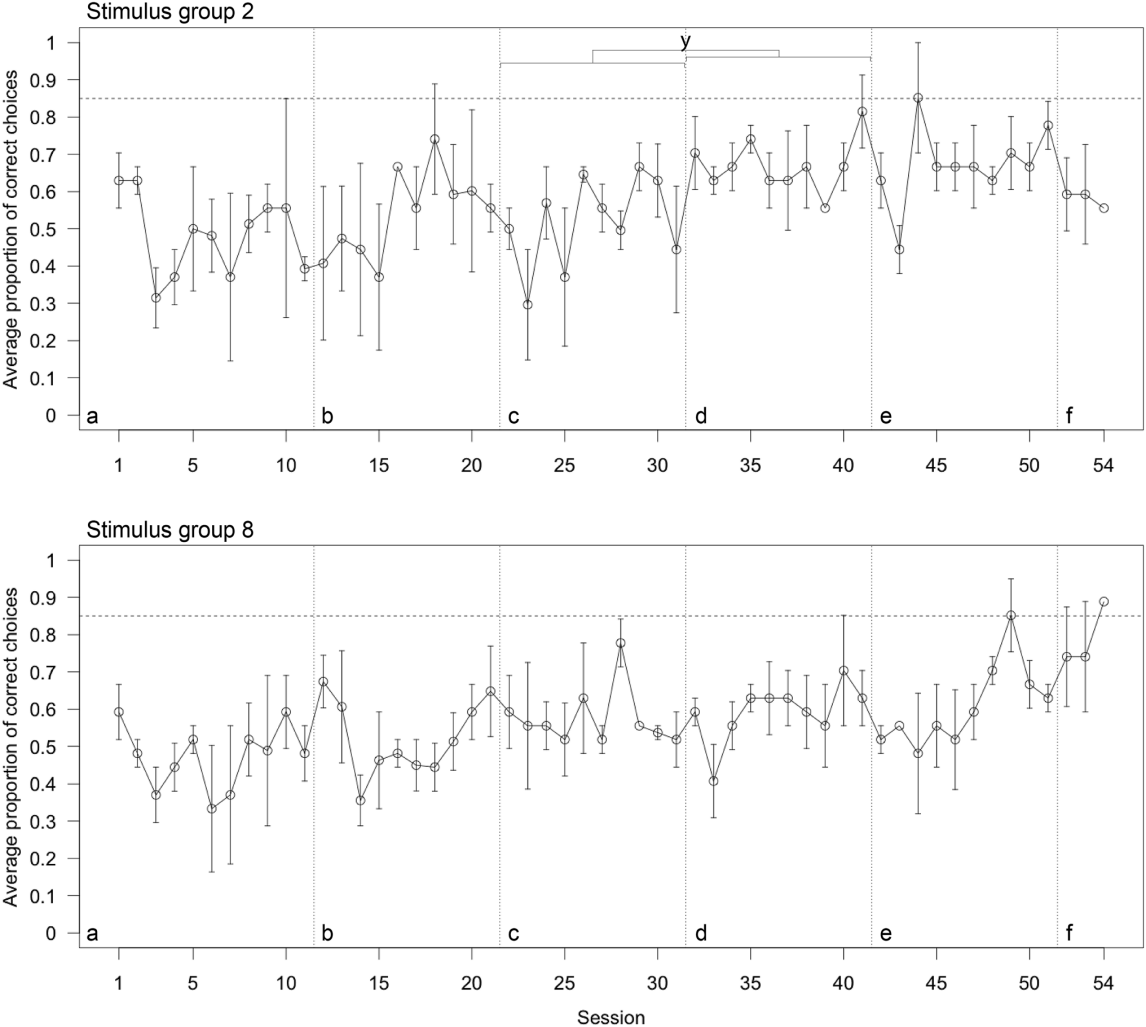
Average proportion of correct choices (± standard error) across sessions of the six lizards tested in the pattern discrimination test split between lizards tested with the stimulus showing two squares as correct (top; *N* = 3) and lizards tested with the stimulus showing eight squares as correct (bottom; *N* = 3). Changes in procedure are indicated with vertical dashed lines: **a** Unchanged original procedure. **b** the target card was replaced with a card showing the correct pattern. **c** Additional target training with the correct card. **d** Reduced physical contact with the lizard. **e** Cleaning of the cue cards with ethanol was stopped. **f** Replacement of the incorrect card with a plain grey card. The horizontal dashed line indicates the learning criterion of 8/9 correct choices or better in each of two consecutive sessions. y—significant difference (confidence intervals—CIs—not crossing 0). Created using R base plot and modified using Adobe Illustrator 2021

None of the lizards reached the learning criterion in the first 11 sessions of the pattern discrimination test. Thereafter, changes were implemented to try and find out possible factors causing the poor performance. Replacing the target grey card (used in the first trial of each session) with a card depicting the correct stimulus (change 1, Fig. 4a, b) did not improve choice performance (GLMM, Table 2 column A–B). Additional training with the card showing the correct stimulus (change 2, Fig. 4b, c) also did not improve performance (GLMM, Table 2 column B–C). Reducing physical contact with the lizards (change 3, Fig. 4c, d) did improve choice performance in the group tested with two as the correct stimulus but not in the lizards tested with eight as the correct stimulus (GLMM, Table 2 column C–D). Reducing contact did not lead to a change in the learning slope (rate of change across sessions) in either group (GLMM, Table 2 column C–D). Not cleaning cue cards with ethanol (change 4, Fig. 4d, e) to facilitate the accumulation of scent also did not improve choice performance (GLMM, Table 2 column D–E). Replacing the incorrect card with an empty grey card (change 5, Fig. 4e, f) did not improve choice performance of lizards trained with the card showing two squares as correct as well as lizards trained with the card showing eight squares as correct (GLMM, Table 2 column E–F). Estimates indicate, however, that the last change had a positive influence on the lizards from stimulus group 8 (GLMM, estimate_intercept_ = 2.285; Fig. 4f) while it had the opposite influence on the lizards from stimulus group 2 (GLMM, estimate_intercept_ = − 0.735; Fig. 4f). Two of the three lizards tested with eight as the correct stimulus reached the learning criterion after the last change was implemented (Supplementary Material Fig. S1). None of the lizards tested with two as the correct stimulus reached the learning criterion (Supplementary Material Fig. S2). Across 51 sessions lizards choice performance only minimally and non-significantly improved (GLMM, estimate = 0.243, CI_low_ = − 0.435, CI_up_ = 0.900). SVL (GLMM, estimate = − 0.191, CI_low_ = − 0.598, CI_up_ = 0.201) and temperature (GLMM, estimate = − 1.524, CI_low_ = − 4.516, CI_up_ = 1.308) were negatively and non-significantly associated with choice performance.

**Table 2:** Estimates and upper and lower 95% confidence interval (95% CI) calculated by the Bayesian generalised linear mixed models used to analyse changes in choice performance (1—correct, 0—incorrect) across changes in testing procedure (A–F) for both stimulus groups together and separated into stimulus group 2 and 8

		A–B	B–C	C–D	D–E	E–F
Both stimulus group 8 and 2						
Intercept	Estimate	0.294	0.080	**0.344**	0.043	0.531
	95% CI	− 0.016 to 0.591	− 0.229 to 0.388	**0.036 to 0.638**	− 0.257 to 0.343	− 2.038 to 2.971
Slope	Estimate	0.108	− 0.061	0.110	**0.165**	− 0.018
	95% CI	− 0.209 to 0.404	− 0.377 to 0.266	− 0.209 to 0.410	**0.146 to 0.476**	− 2.005 to 1.936
Stimulus group 2						
Intercept	Estimate	0.378	− 0.158	**0.645**	0.002	− 0.735
	95% CI	− 0.065 to 0.821	− 0.627 to 0.296	**0.209 to 1.100**	− 0.426 to 0.433	− 4.111 to 2.573
Slope	Estimate	0.181	− 0.012	− 0.041	0.156	− 0.451
	95% CI	− 0.276 to 0.622	− 0.496 to 0.460	− 0.514 to 0.408	− 0.282 to 0.605	− 3.013 to 2.336
Stimulus group 8						
Intercept	Estimate	0.224	0.275	0.085	0.081	2.285
	95% CI	− 0.188 to 0.639	− 0.156 to 0.688	− 0.343 to 0.492	− 0.334 to 0.514	− 1.632 to 6.364
Slope	Estimate	0.041	− 0.078	0.229	0.179	0.724
	95% CI	− 0.381 to 0.448	− 0.522 to 0.353	− 0.200 to 0.661	− 0.259 to 0.612	− 2.458 to 3.804

We assumed statistical significance if confidence intervals did not cross 0. Significant results are highlighted in bold

#### Latency to choice

**Fig. 5:**
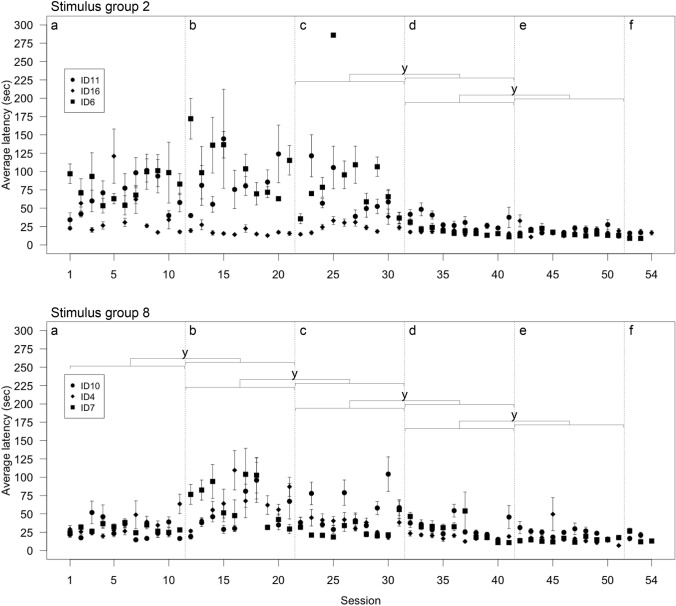
Average latency in sec (± standard error) across sessions of the six lizards tested in the pattern discrimination test split between lizards tested with the stimulus showing two squares as correct (top; *N* = 3) and lizards tested with the stimulus showing eight squares as correct (bottom; *N* = 3). Each lizard’s average latency (± standard error) for each session is plotted with a unique symbol to identify data stemming from the same individual across sessions. Changes in procedure are indicated with vertical dashed lines: **a** Unchanged original procedure. **b** the target card was replaced with a card showing the correct pattern. **c** Additional target training with the correct card. **d** Reduced physical contact with the lizard. **e** Cleaning of the cue cards with ethanol was stopped. **f** Replacement of the incorrect card with a plain grey card. y—significant difference (confidence intervals—CIs—not crossing 0). Created using R base plot and modified using Adobe Illustrator 2021

Replacing the target grey card with a card depicting the correct stimulus (change 1, Fig. 5a, b) did not change latency in lizards tested in stimulus group 2 but increased latency in lizards from stimulus group 8 (GLMM, Table 3 column A–B). The slope (rate of change across sessions) stayed the same (GLMM, Table 3 column A–B). In lizards tested with two as the correct stimulus, no reduction in latency occurred after additional training with the card showing the correct stimulus (change 2, Fig. 5b, c) while lizards tested with eight as the correct stimulus reduced latency; again, the slope did not change (GLMM, Table 3 column B–C). After reducing physical contact with the lizards (change 3, Fig. 5c, d), we found that individuals from both groups reduced latency to choice significantly (GLMM, Table 3 column C–D). We also found no change in slope in the group tested with two squares (GLMM, Table 3 column C–D) but found that latency significantly decreased across sessions in lizards from stimulus group 8 (GLMM, Table 3 column C–D). Not cleaning cue cards with ethanol (change 4, Fig. 5d, e) reduced latency further in both groups (GLMM, Table 3 D–E). Lastly, latency did not change after replacing the incorrect card with an empty grey card (GLMM, Table 3 column E–F, change 5, Fig. 5e, f). Across 51 sessions the lizards’ latency to choice only minimally and non-significantly decreased (GLMM, estimate = − 0.068, CI_low_ = − 0.508, CI_up_ = 0.247). SVL (GLMM, estimate = 0.104, CI_low_ = − 0.041, CI_up_ = 0.247) and temperature (GLMM, estimate = 0.401, CI_low_ = − 0.640, CI_up_ = 1.457) were positively and non-significantly associated with latency.

**Table 3:** Estimates and upper and lower 95% confidence interval (95% CI) calculated by the Bayesian generalised linear mixed model used to analyse changes in latency to make a choice (1—correct, 0—incorrect) across changes in testing procedure (A–F) for both stimulus groups together and separated into stimulus group 2 and 8

		A–B	B–C	C–D	D–E	E–F
Both stimulus group 8 and 2						
Intercept	Estimate	0.238	− 0.176	**− 0.559**	**− 0.288**	− 0.174
	95% CI	0.155 to 0.321	− 0.262 to − 0.095	**− 0.642 to − 0.479**	**− 0.369 to − 0.209**	− 0.792 to 0.464
Slope	Estimate	− 0.006	− 0.010	**− 0.221**	**0.144**	0.007
	95% CI	− 0.089 to 0.076	− 0.100 to 0.077	**− 0.303 to − 0.136**	**0.062 to 0.226**	− 0.493 to 0.495
Stimulus group 2						
Intercept	Estimate	− 0.110	− 0.019	**− 0.771**	**− 0.245**	0.524
	95% CI	− 0.236 to 0.019	− 0.150 to 0.111	**− 0.898 to − 0.647**	**− 0.367 to − 0.124**	− 0.436 to 1.488
Slope	Estimate	− 0.106	− 0.017	− 0.130	**0.137**	0.588
	95% CI	− 0.237 to 0.021	− 0.159 to 0.118	− 0.258 to 0.002	**0.009 to 0.261**	− 0.193 to 1.341
Stimulus group 8						
Intercept	Estimate	0.626	**− 0.382**	**− 0.423**	**− 0.328**	− 0.848
	95% CI	0.519 to 0.734	**− 0.489 to − 0.272**	**− 0.533 to − 0.319**	**− 0.434 to − 0.220**	− 1.708 to 0.009
Slope	Estimate	− 0.002	− 0.010	**− 0.261**	**0.153**	− 0.557
	95% CI	− 0.114 to 0.105	− 0.126 to 0.105	**− 0.376 to − 0.151**	**0.043 to 0.267**	− 1.215 to 0.135

We assumed statistical significance if confidence intervals did not cross 0. Significant results are highlighted in bold

## Discussion

Our study demonstrates a new protocol in which we successfully trained lizards to approach and touch a target card to receive a food reward. The low variability in the sessions taken to reach the performance criteria over the last two steps of behavioural approximation (Pre2 and Pre3) across all tested individuals, is evidence that this is a robust procedure. We were able to use this trained behaviour in a simultaneous two-choice discrimination between light and dark blue (pilot) and between a card showing two, and a card showing eight, squares (pattern discrimination). Although lizards acquired the colour discrimination within 6–10 sessions, they were unable to discriminate between the two patterns (for 51 sessions). Some individuals, all from stimulus group 8, learnt the discrimination only after we replaced the incorrect stimulus with a blank grey card; these individuals then learnt within two to three sessions (a minimum of two sessions was required to reach criterion). Moreover, reducing physical contact considerably reduced latency in some lizards and had a significant effect on choice performance in stimulus group 2. This significant reduction in latency shows how seemingly already well-adjusted and habituated individuals that rarely show fear behaviour (fleeing and hiding) in the presence of a human might still experience stress during testing that can increase latency measures and confound inferences drawn if such measures are used as evidence for learning. We acknowledge that a drawback of our protocol is that a researcher has to be present to test each individual animal as opposed to setting up a batch of animals to be tested remotely (i.e. using filming). In experiments in which a higher level of control needs to be exerted by a researcher (e.g. ability to immediately remove a stimulus; e.g. Szabo et al. 2021c) or many trials have to be given over a short time span, our method is preferable.

Stepwise approximation has previously been used in lizard research to train animals to open lids to receive a food reward (e.g. Damas-Moreira et al. 2018; Leal and Powell 2012; Noble et al. 2014; Riley et al. 2018; Whiting et al. 2018). In zoos, target training is generally used to facilitate husbandry procedures, behavioural enrichment, health checks and medical interventions (e.g. Hellmuth et al. 2012). Here, we provide detailed data on the progress of our lizards to acquire the desired behaviour of approaching and touching one of two stimulus cards. We used a well-established procedure of rewarding the desired behaviour until it was shown consistently. Although the number of training sessions varied more in the first step of training (Pre1), as soon as lizards had associated touching the card with food, they generally needed close to, or the minimum number, of sessions to reach our performance criterion (Pre2 and Pre3). Our data show, therefore, that the procedure we used was well suited to target train gidgee skinks.

In the pilot, we chose colour as the relevant cue because our results from a previous study (Szabo et al. 2021b) showed that gidgee skinks were able to learn to discriminate between light and dark blue stimuli. The pilot was designed to test if our training and test procedure, including our new learning criterion, were sufficient to facilitate and detect learning. As our results show, learning progressed as expected and the robustness of our learning criterion was confirmed using a reversal trial. We found the expected drop in performance in the reversal which we could not detect in the previous study using the same stimuli (Szabo et al. 2021b).

Our findings from the pattern discrimination task revealed interesting new insights into visual stimulus processing in lizards. Although to the human observer the two stimuli of two or eight squares on a grey background were easily distinguishable our results show that lizards were not able to make this discrimination. We propose that this issue was caused by the overlap between the two visual patterns: the two middle squares had the exact same size and were in the exact same position in both cards. This overlap, i.e., a common feature in both cards, might have prompted our lizards to generalise across cards which could have interfered with their discrimination (Astley and Wasserman 1992). After we replaced the incorrect stimulus with a blank grey card, performance shown by lizards from stimulus group 8 increased while it decreased in lizards from stimulus group 2. The difference in response between the two groups can also be explained by generalisation. While the card with eight squares showed little background, the card with two squares showed a lot of background in the same grey as the blank grey card. Therefore, lizards from stimulus group 2 might, again, have generalised but instead of the squares they generalised based on the amount of background shown. Why lizards from stimulus group 2 decreased performance is not clear. It is, therefore, necessary to implement further tests to draw accurate conclusions about why our lizards had problems discriminating the two patterns. For example, adding extra information to each stimulus that increases their distinctiveness, such as changing the colour of the central squares, might overcome any issues caused by stimulus generalisation, possibly improving performance (Shettleworth 2010). Furthermore, instead of presenting the two squares in the exact same position in both cards, we could move them to novel positions. Another option would be to present the two squares at a position corresponding with two of the outer six squares within the pattern of eight. This could help establish if the overlap constrained learning. Finally, testing more lizards would also help to establish if this is a general issue in this species or only present in some individuals.

Two main reasons for our lizards’ poor performance, low visual acuity and issues with attention, can be ruled out. Although there are no studies looking into gidgee skinks visual acuity, these lizards have shown learning in a previous experiment in which they had to discriminate between shapes in a similar simultaneous two-choice discrimination task (Szabo et al. 2021b). Even if lizards were unable to perceive each single square within a card used in the current study, the squares took up very different amounts of space on each card. Lizards could have either relied on overall luminance/chromatic contrast or the size of the black portion on the card to learn the discrimination (or, alternatively, how much grey background was visible). The fact that some lizards did eventually reach criterion after we replaced the incorrect card, together with the results from our previous study (Szabo et al. 2021b), does suggest that lizard visual acuity is good enough to perceive single squares and that stimulus generalisation was responsible for their poor performance.

Similarly, a failure to attend to the correct stimulus features seems unlikely because performance did improve across sessions, possibly because their perceptual system became more attuned to the patterns. When the discriminability of the stimuli was increased, some lizards immediately reached the learning criterion. Furthermore, the results from our previous work (Szabo et al. 2021b) showed that these lizards are able to learn a discrimination based on cues incorporating multiple features (colour and shape simultaneously) of which only one feature set was reliably correlated with reinforcement, while the other was not. Some lizards even demonstrated learnt irrelevance as their choice behaviour was unaffected by changes to the irrelevant feature set (Szabo et al. 2021b). It seems, therefore, most likely that our lizards’ issue was due to overlapping central representation and associated processing difficulties.

Apart from the striking inability of our lizards to learn to discriminate the presented patterns, we uncovered important new insights regarding latency that can help improve future studies of learning in lizards. Reducing physical contact with the test animals had a strong positive effect on latency. Although some individuals were seemingly less affected by direct physical contact (low latency from the beginning), other individuals showed a positive reaction (shorter latency) when this physical contact was removed. These lizards had been kept in the lab and cared for by the same researcher for over a year and had seemingly habituated well to captivity and different testing procedures. Our results, however, show that even seemingly well habituated animals can still experience stress during testing. Although reducing physical contact had only small effects on choice, stronger effects might be expected in animals less well habituated to the experimenter. If, in future studies, sample size is increased, reductions in latency can be a major benefit allowing researchers to test more individuals in the available timeframe which will positively influences statistical power. Importantly, if latency is used as a measure of learning (e.g. Amiel and Shine 2012; Chung et al. 2017; Cooper et al. 2019) researchers need to be aware of how the testing procedure might affect latency measures. If individual lizards experience testing as more stressful and respond slowly, results might be negative, concluding that lizards did not learn when in fact the procedure was not suitable and confounded the results.

## Conclusion

We provide a new protocol to test lizards in a two-choice discrimination task. We show that even in the case of low average performance, lizards can be tested with more than 1–3 trials per day. This frequency of testing is currently widely adopted (e.g. Bezzina et al. 2014; Clark et al. 2014; Damas-Moreira et al. 2018; Leal and Powell 2012; Munch et al. 2018; Noble et al. 2014; Qi et al. 2018; Riley et al. 2018; Szabo et al. 2018; 2019a; b; 2021b; Szabo and Whiting 2020; Whiting et al. 2018). The protocol involves operant conditioning and successive approximation to condition lizards to touch a cue card. This behaviour can then be applied in a discrimination task and as our results demonstrate, lizards consistently performed this behaviour across many hundreds of trials. We also showed that reducing physical contact with a study animal can significantly reduce testing time. We suggest this is a robust procedure that can be used in lizard species that are able to consume numerous small food rewards in a single day. Although we do not suggest a one-size-fits-all procedure, we do advocate for consistency, when possible. If more researchers use the same approach, we will be well placed to conduct comparative studies which will greatly improve our understanding of lizard cognition and the evolution of cognition more broadly.

## Supplementary Information

Below is the link to the electronic supplementary material.

Supplementary Video M1 (MP4 378028 KB)

Supplementary figures (DOCX 671 KB)

## Data Availability

All data generated during this study are available on the Open Science Framework (OSF). 10.17605/OSF.IO/SDUX7.

## Code availability

All code used for analysis in R (version 4.0.3, R Core Team, 2020) are available on the Open Science Framework (OSF). 10.17605/OSF.IO/SDUX7.
