# Supplementary material for: A new protocol for investigating visual two-choice discrimination learning in lizards
Source: Anim Cogn. 2022 Feb 6;25(4):935–50. doi: 10.1007/s10071-022-01603-x (PMC9334418; doi:10.1007/s10071-022-01603-x)
Supplement: Supplementary file 2 — Supplementary figures (DOCX 671 KB) [file 10071_2022_1603_MOESM2_ESM.docx]

# Supplementary material

To

“An new protocol for investigating visual two-choice discrimination learning in lizards”

By Birgit Szabo^a,b^ * and Martin J. Whiting^a^

Animal Cognition

^a^ Department of Biological Sciences, Macquarie University, Sydney, Australia

^b^ Division of Behavioural Ecology, Institute of Ecology and Evolution, University of Bern, Bern, Switzerland

*Correspond to: Birgit Szabo, Division of Behavioural Ecology, University of Bern, 3032 Bern, Switzerland; email: [birgit.szabo@gmx.at](mailto:birgit.szabo@gmx.at)

## Supplementary Figures


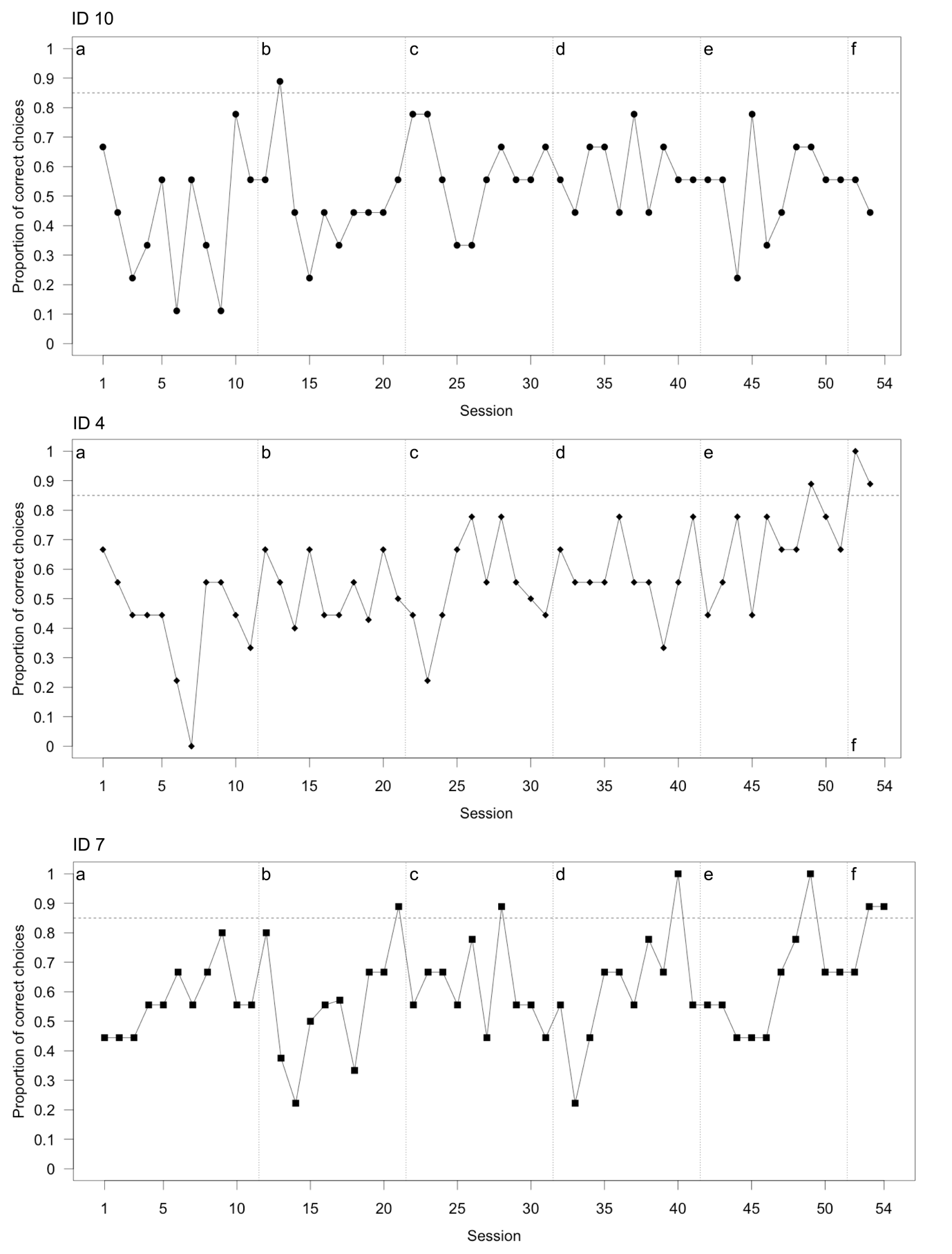


**Fig. S1** Proportion of correct choices across sessions of the three lizards (ID 10, ID 4, and ID 7) tested in the pattern discrimination with eight as the correct cue. Symbols correspond to symbols in Fig. 5 from the main text. Changes in procedure are indicated with vertical dashed lines: a) Unchanged original procedure. b) the target card was replaced with a card showing the correct pattern. c) Additional target training with the correct card. d) Reduced physical contact with the lizard. e) The addition of scent on the correct card. f) Replacement of the incorrect card with a plain grey card. Created using R base plot and modified using Adobe Illustrator 2021


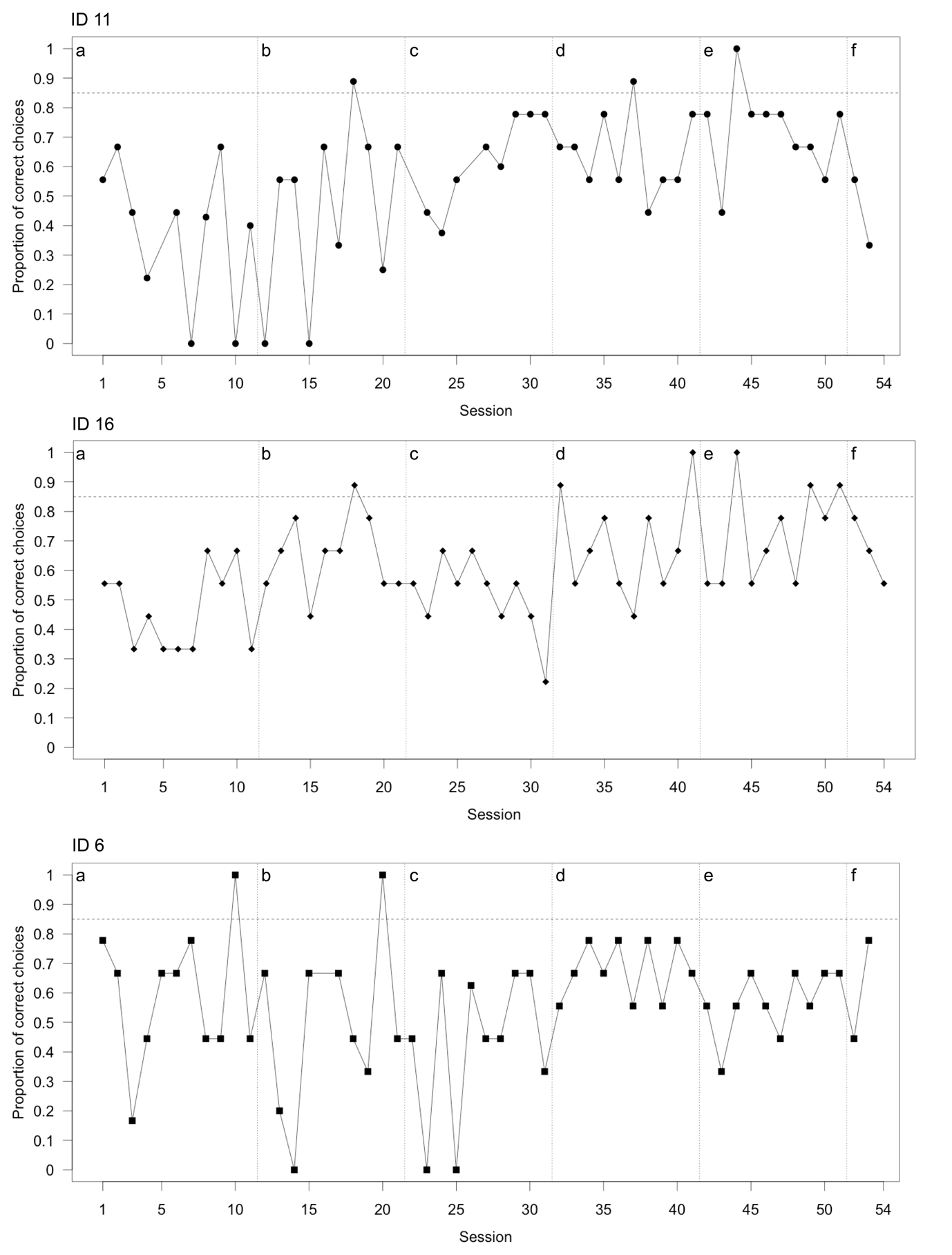


**Fig. S2** Proportion of correct choices across sessions of the three lizards (ID 11, ID 16, and ID 6) tested in the pattern discrimination with two as the correct cue. Symbols correspond to symbols in Fig. 5 in the main text. Changes in procedure are indicated with vertical dashed lines: a) Unchanged original procedure. b) the target card was replaced with a card showing the correct pattern. c) Additional target training with the correct card. d) Reduced physical contact with the lizard. e) The addition of scent on the correct card. f) Replacement of the incorrect card with a plain grey card. Created using R base plot and modified using Adobe Illustrator 2021
